# Pentaethylenehexamine-Loaded Hierarchically Porous Silica for CO_2_ Adsorption

**DOI:** 10.3390/ma9100835

**Published:** 2016-10-15

**Authors:** Changchun Ji, Xin Huang, Lei Li, Fukui Xiao, Ning Zhao, Wei Wei

**Affiliations:** 1 State Key Laboratory of Coal Conversion, Institute of Coal Chemistry, Chinese Academy of Sciences, 27th South Taoyuan Road, Taiyuan 030001, China; jichangchun@sxicc.ac.cn (C.J.); huangxin11987@163.com (X.H.); lilei@sxicc.ac.cn (L.L.); xiaofk@sxicc.ac.cn (F.X.); 2 University of Chinese Academy of Sciences, Beijing 100049, China; 3 National Engineering Research Center for Coal-Based Synthesis, Institute of Coal Chemistry, Chinese Academy of Sciences, Taiyuan 030001, China; 4 Center for Greenhouse Gas and Environmental Engineering, Shanghai Advanced Research Institute, Chinese Academy of Sciences, Shanghai 201203, China

**Keywords:** CO_2_ capture, hierarchically porous silica, pentaethylenehexamine

## Abstract

Recently, amine-functionalized materials as a prospective chemical sorbent for post combustion CO_2_ capture have gained great interest. However, the amine grafting for the traditional MCM-41, SBA-15, pore-expanded MCM-41 or SBA-15 supports can cause the pore volume and specific surface area of sorbents to decrease, significantly affecting the CO_2_ adsorption-desorption dynamics. To overcome this issue, hierarchical porous silica with interparticle macropores and long-range ordering mesopores was prepared and impregnated with pentaethylenehexamine. The pore structure and amino functional group content of the modified silicas were analyzed by scanning electron microscope, transmission electron microscope, N_2_ adsorption, X-ray powder diffraction, and Fourier transform infrared spectra. Moreover, the effects of the pore structure as well as the amount of PEHA loading of the samples on the CO_2_ adsorption capacity were investigated in a fixed-bed adsorption system. The CO_2_ adsorption capacity reached 4.5 mmol CO_2_/(g of adsorbent) for HPS−PEHA-70 at 75 °C. Further, the adsorption capacity for HPS-PEHA-70 was steady after a total of 15 adsorption-desorption cycles.

## 1. Introduction

To date, carbon capture and storage (CCS) technology has been established as a main and efficient solution to resolve global warming and climate change issues [1,2,3,4]. For economic storage of CO_2_, to become cost-effective, CO_2_ in a relatively centralized flow is very important. However, the cost of the carbon capture accounts for 70% of the whole CCS process [5]. Currently, CO_2_ capture and separation methods mainly include absorption, membrane separation and solid adsorption, etc. [6,7,8,9,10]. Amine solutions, including monoethanolamine, diethanolamine, diglycolamine, and N-methyldiethanolamine, are widely used for CO_2_ capture or separation [11]. However, energy consumption associated with the process is inherently high because of solvent regeneration and equipment corrosion [12]. Compared with solvent absorption and membrane separation, solid adsorption has many potential advantages for CO_2_ capture, such as high capacity, good selectivity, and easy processing [13]. In the last few years, new solid CO_2_ capture sorbents with excellent performance and cost efficiency have been developed [14,15,16,17,18,19,20,21,22,23]. To improve the CO_2_ selectivity and adsorption capacity, a variety of micro-meso-porous materials loaded with basic nitrogen functionalities have been found to be very promising [24,25,26,27]. The adsorption capacity of carbonaceous materials could obviously increase via the introduction of certain functional groups. Currently, alkali carbonate and various amino functional groups are employed as modification functional groups [24,28] and the modification can be realized on porous materials such as carbon nanotubes, zeolite 13X, β-zeolite, SBA-15, SBA-16, MCM-41, and MCM-48 [29,30,31,32,33,34,35,36]. In 2002, Song et al. first reported that the CO_2_ adsorption capacity based on MCM-41 impregnated with PEI was 3 mmol·g^−1^ under pure CO_2_ and 75 °C [37], which was named as a “molecular basket”. Compared with chemical adsorption, physical adsorption of MCM-41 is negligible. Maroto Valer et al. also prepared a PEI impregnated sorbent with a CO_2_ adsorption capacity of 2.13 mmol·g^−1^ at 75 °C, higher than that of without impregnation [38]. Gargiulo et al. revealed that the pore size can affect the CO_2_ adsorption capacity by comparing the PEI functionalized mesoporous MCM-48 and SBA-15 [39]. Otherwise, pore volume and specific surface area of the sorbents may significantly influence the CO_2_ adsorption-desorption kinetics [40]. As a result, pore expansion agent was applied to improve the pore size and pore volume. Sayari et al. found that higher adsorption capacity could be obtained after reaming MCM-41 [41]. Yan and coworkers also observed that CO_2_ adsorption capacity increased significantly on increasing the pore volume [40]. Ma reported that the SBA-15 (50% (w) PEI) sorbent treated with ammonia at 75 °C showed a CO_2_ adsorption capacity of 3.18 mmol·g^−1^ [42]. Qi et al. also exploited high efficient CO_2_ capture nanocomposite sorbent on the basis of oligomeric amine-functionalized mesoporous capsules [43].

However, these mesoporous materials had relatively low adsorption capacity in flue gas because of the small pore diameter. To mitigate the problem, several research groups initiated works on pore-expanded supports. Siliceous mesostructured cellular foams (MCF) and nonpolar resin HP20 with large pore diameter also showed high adsorption capacity for pure CO_2_. However, the CO_2_ uptake was low for flue gas. Moreover, PEI, mainly used as organic amine for modification, is a large molecule and cannot enter the channel of the porous materials easily. Compared with the PEI, pentaethylenehexamine (PEHA) with higher amine group content, a more excellent thermal stability as well as lower toxicity can be achieved for modification [44,45,46,47].

In this work, PEHA-loaded hierarchically porous silica with macropores and long-range ordering mesopores were prepared. Compared with MCM-41 and SBA-15 with two-dimensional channels of mesoporous, silica with a hierarchical pore structure has not only a large and adjustable macroporous that favors adsorption, but also a tunable mesoporous and a larger entrance for mass transfer. As a result, the CO_2_ adsorption capacity is improved. It was also found that the temperature and the PEHA amount supported on the porous silica obviously affected the sorption behavior.

## 2. Experimental

### 2.1. Material Preparation

#### 2.1.1. Preparation of the Support

The general synthesis protocol for hierarchical porous silica (HPS) followed a sol-gel method by Smatt et al. [48]. Typically, tetraethoxysilane (TEOS, Sigma-Aldrich, St. Louis, MO, USA) was added to a mixture of poly(ethylene glycol) (PEG, Sigma-Aldrich, Mw 20,000, St. Louis, MO, USA) dissolved in HNO_3_ aqueous solution, and the sol was then stirred at 25 °C to form a clear solution. After that, cetyltrimethylammonium bromide (CTAB, Sinopharm chemical Reagent Co., Ltd., Shanghai, China) was added and stirred to completely dissolve the surfactant. The as-obtained sol was placed to form gel and further aged for 48 h at 40 °C. In order to obtain large textural pores, the gel was kept in 1 M NH_4_OH solution for 9 h at 90 °C. The crystals used 0.1 M HNO_3_ solution to acidify and were then washed with ethanol. Finally, the gel was dried at 60 °C for 3 days followed by calcination at 550 °C for 5 h to obtain the HPS.

#### 2.1.2. Preparation of the Adsorbent

The PEHA-modified HPS support was fabricated via wet impregnation. Typically, the appropriate amount of PEHA was dissolved in 5 g methanol under stirring at 40 °C for 30 min and then 0.5 g porous silica was added. Finally, the sorbent was obtained by continuously stirring the resultant slurry for 8 h and then drying at 75 °C for 8 h. The as-prepared sorbent was denoted as HPS-PEHA-x and x represented the PEHA weight percentage.

### 2.2. Measurements of CO_2_ Adsorption Capacity

Adsorption and regeneration processes of CO_2_ were carried out in a fixed-bed quartz reactor. Typically, sample (1.0 g, 20-40 mesh) was packed into the reactor and heated under a flow of 48 mL/min in argon at 100 °C for 2 h. Besides, after cooling to adsorption temperature (30, 50, 70, 75, and 90 °C), the simulated flue gas including CO_2_ (10 vol %) and N_2_, were introduced into the reactor. The CO_2_ concentration of outlet gases was detected every 10 s via a gas analyzer (Vaisala, Helsinki, Finland).

### 2.3. Characterization

The X-ray diffraction (XRD) patterns were analyzed by a D8 Advance X-ray diffractometer (Rigaku, Tokyo, Japan) with Cu Kα adiation (λ = 0.154046 nm).

N_2_ adsorption/desorption isotherms were conducted on a ASAP-2020 instrument (Micromeritics, Atalanta, GA, USA).

The scanning electron microscopy (SEM) images of materials were carried out on a JSM-7001F instrument (JEOL, Beijing, China) at 5 kV.

Transmission electron microscopy (TEM) images of samples were obtained on a JEOL 2010 microscope (JEOL, Beijing, China) operated at 200 kV.

A Nicolet Magna-II550 spectrometer (Madison, WI, USA) was used to obtain the Fourier transform infrared spectroscopy (FT-IR) of the amine-attached hierarchically porous silica.

The diffuse reflection infrared Fourier transform spectroscopy (DRIFTS) was conducted on a Nicolet Magna-II 550 spectrometer. 

Elemental analysis was recorded using a vario Macro cube instrument (Elementar, Germany) to determine the N content.

## 3. Results and Discussion

### 3.1. Characterization of Adsorbents

**Scheme 1:**
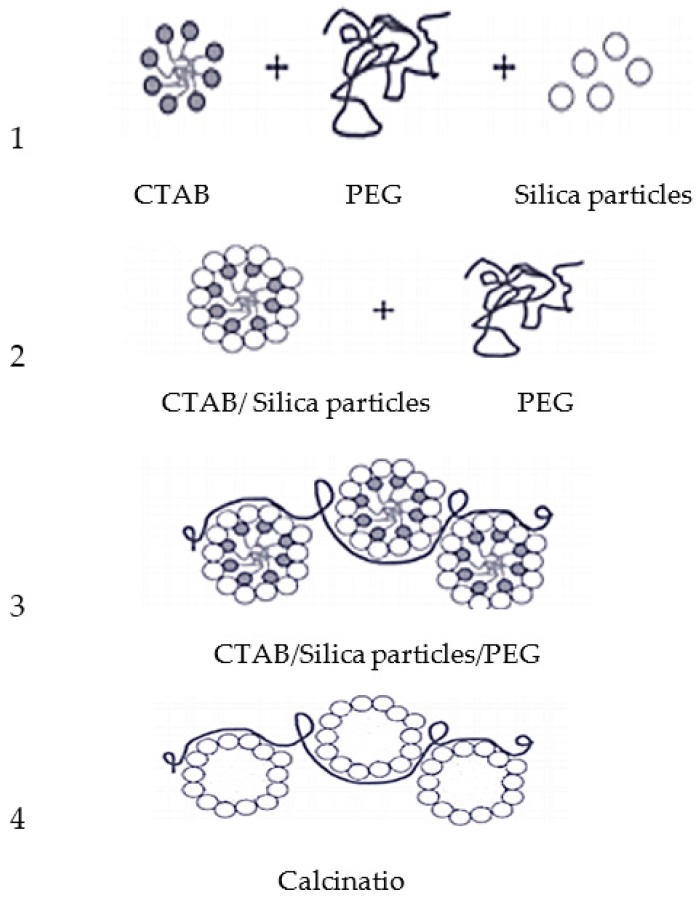
Synthesis steps of the hierarchical porous silica sorbents.

**Figure 1:**
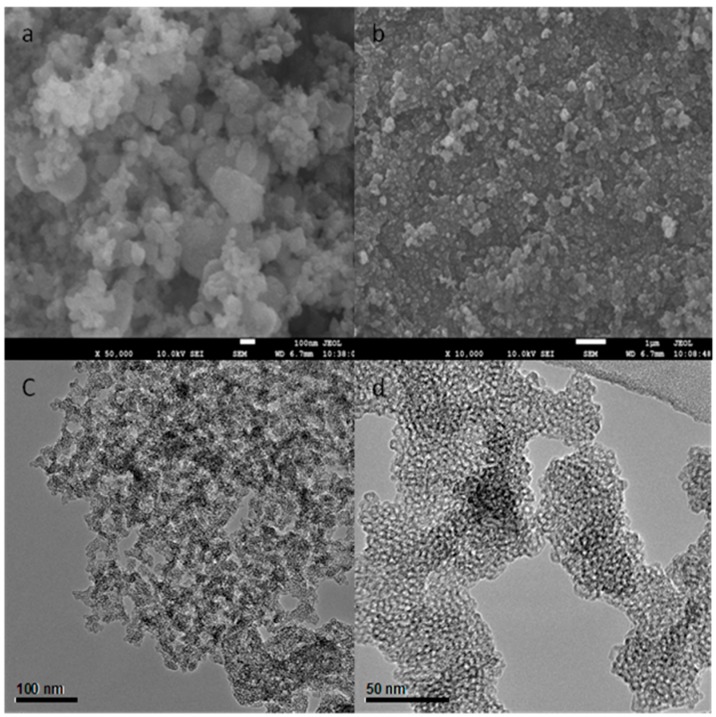

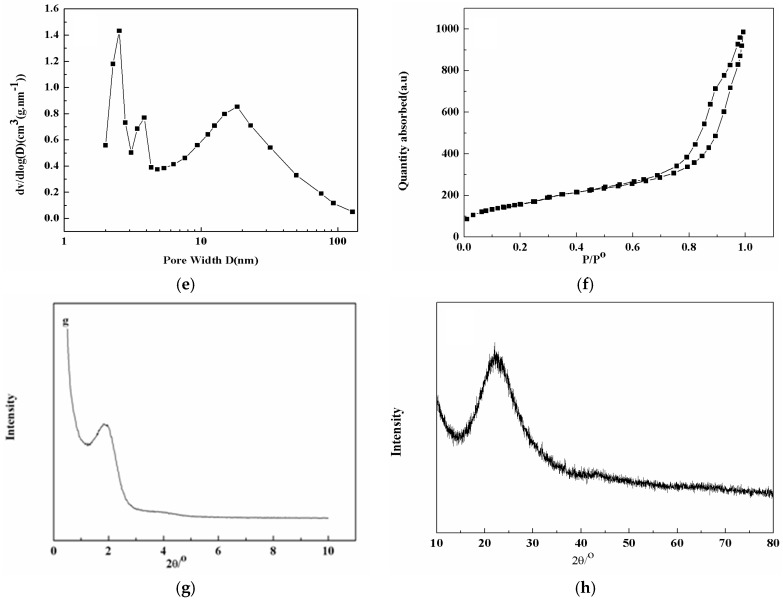
(**a**) SEM image of HPS; (**b**) SEM image of HPS; (**c**) TEM image HPS; (**d**) TEM image HPS; (**e**) Pore size distribution of HPS; (**f**) N_2_ adsorption/desorption isotherms of HPS; (**g**) Small-angle XRD of HPS; (**h**) Wide-angle XRD of HPS.

According to Smatt et al. [48], the material synthesis can be divided into four steps (Scheme 1). The formation of HPS with multimodal porosity is realized by applying the surfactant CTAB and PEG as templates. After roasting, supports with mesoporous and macroporous were obtained. Typically, monolithic rod diameters about 1–3 cm are observed as in Figure 1a,b. The silica monoliths possess open 3-D network macropores (Figure 1c,d) [48,49,50]. The material also contains interparticle macropores and intraparticle wormlike mesopores, as shown in Figure 1c,d. Figure 1e,f shows the adsorption and desorption isotherm and pore size distribution of the as-prepared material. Two distinct peaks centered at approximately 2–4 nm and 8–70 nm can be found [50,51,52]. Low-angle reflection peaks centered at 1.8° (Figure 1g) can also be observed which confirm the ordered mesopores [53,54,55]. Intense and wide peaks centered at around 23° (Figure 1h) are also found corresponding to the amorphous SiO_2_ that constitutes the macropores. The HPS could not only offer a large specific surface area but also favored an access of active sites.

**Figure 2:**
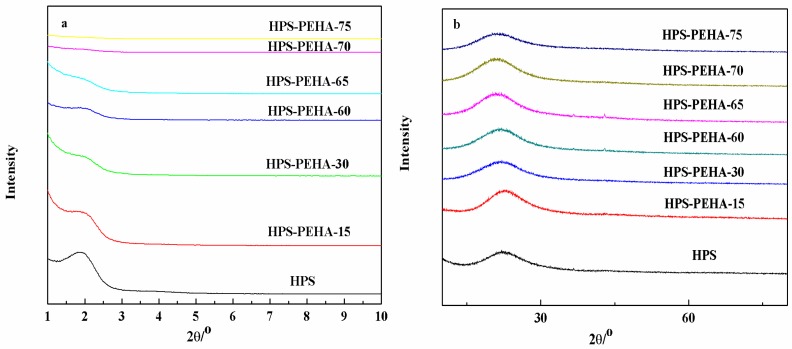
XRD profiles of HPS and HPS-PEHA-x adsorbents: (**a**) small-angle XRD of HPS and HPS-PEHA-x adsorbents; (**b**) wide-angle XRD of HPS and HPS-PEHA-x adsorbents.

The XRD patterns of HPS and HPS-PEHA-x adsorbent are shown in Figure 2. For all HPS-PEHA-x adsorbents, the positions of the major peaks are basically the same, which suggests that the HPS structure could still be retained after loading the PEHA. However, the diffraction peak intensity decreases with increasing PEHA content. When the PEHA loading is as high as of 70 wt %, the mesopores are almost all filled due to the pore-filling effect of PEHA.

**Figure 3:**
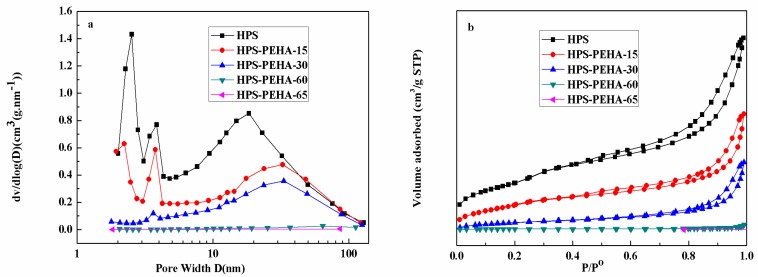
(**a**) Pore size distributions of HPS and HPS-PEHA-x adsorbents; (**b**) N_2_ adsorption-desorption isotherms of HPS and HPS-PEHA-x adsorbents.

Figure 3 shows the pore size distributions and N_2_ adsorption/desorption isotherms of the adsorbents. The specific surface areas and pore volumes are listed in Table 1. With the increase of the PEHA amount, mesopores are filled while some macropores are maintained which is consistent with the results of average pore diameter (Table 1). When increasing the PEHA loading to 65%, the average pore diameter of the material increases to 23.90 nm. Although the N_2_ adsorption capacity decreases as the PEHA amount increases, the isotherms shape is similar, suggesting a decrease of the BET surface area and the pore volume e.g., BET surface area and pore volume of HPS are 544 m^2^/g and 1.00 cm^3^/g, respectively, which decrease to 302 m^2^/g and 0.6 cm^3^/g for PEHA loading of 15% and 2 m^2^/g and 0.00 cm^3^/g for PEHA loading of 65%, respectively.

**Figure 4:**
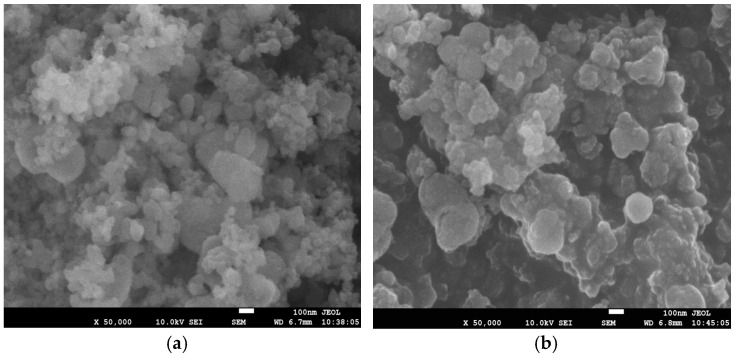

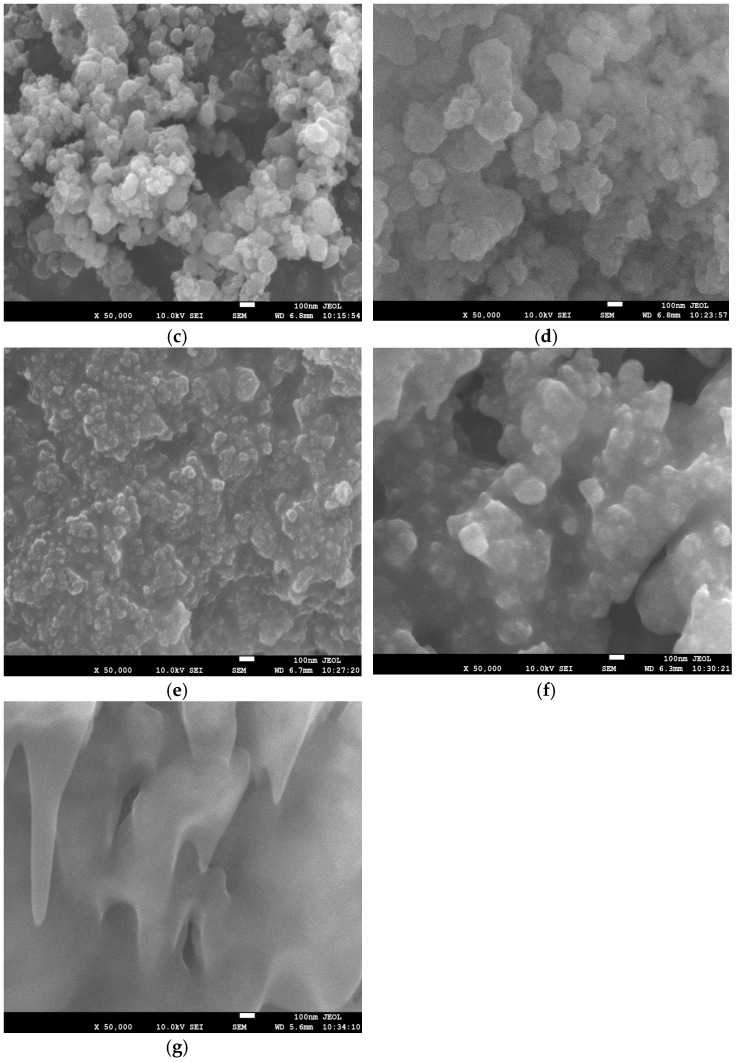
SEM images of of HPS and HPS-PEHA-x adsorbents: (**a**) HPS; (**b**) HPS-PEHA-15; (**c**) HPS-PEHA-30; (**d**) HPS-PEHA-60; (**e**) HPS-PEHA-65; (**f**) HPS-PEHA-70; (**g**) HPS-PEHA-75.

**Figure 5:**
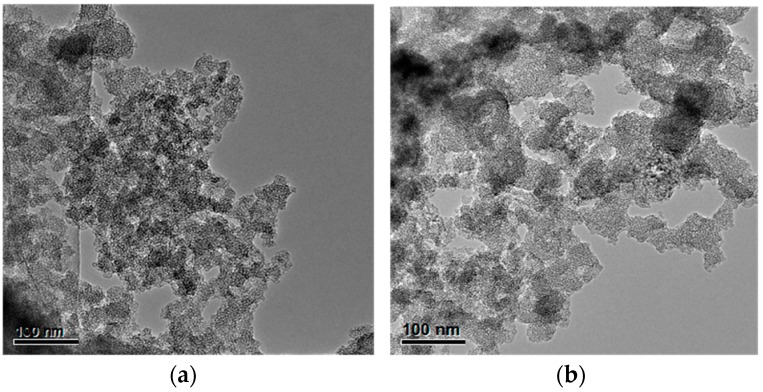

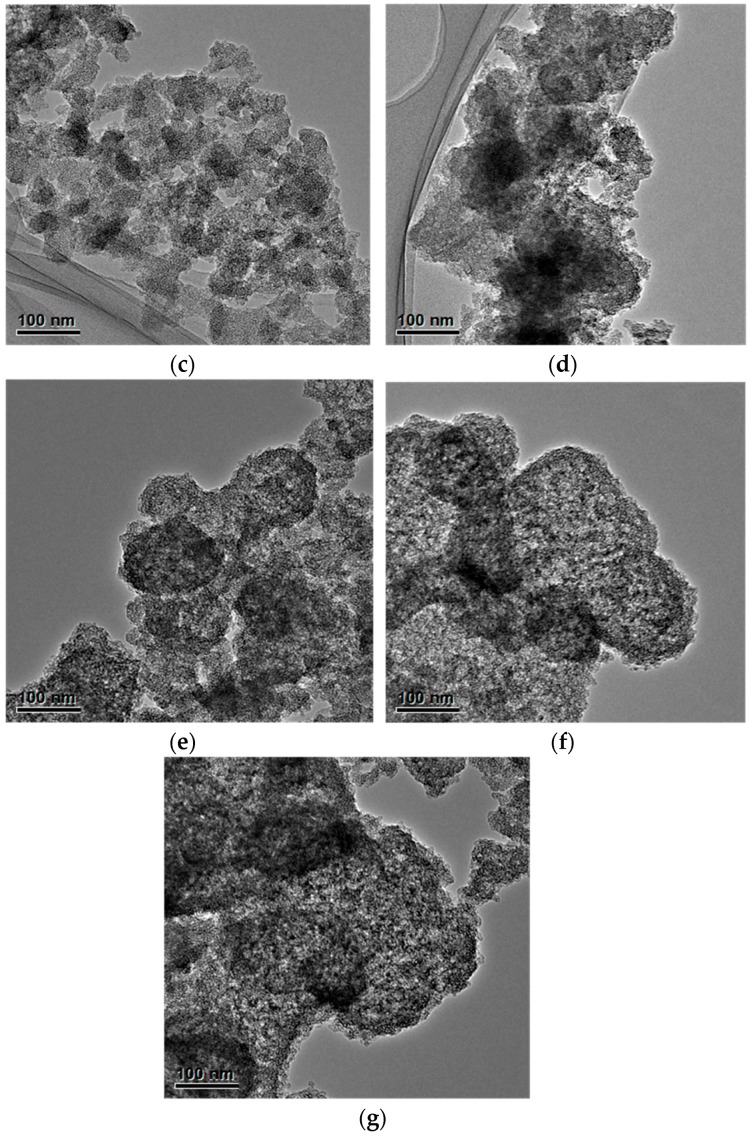
TEM images of of HPS and HPS-PEHA-x adsorbents: (**a**) HPS; (**b**) HPS-PEHA-15; (**c**) HPS-PEHA-30; (**d**) HPS-PEHA-60; (**e**) HPS-PEHA-65; (**f**) HPS-PEHA-70; (**g**) HPS-PEHA-75.

Figure 4 and Figure 5 present the SEM and TEM images of the materials after PEHA loading. Only slight differences are observed with the low PEHA loading (Figure 4c and Figure 5c). When the loading of PEHA is as high as 65%, most of the mesopores are covered with PEHA while some macropores still exist (Figure 4e and Figure 5e). However, on the content of PEHA loading further increasing to 75%, all the pores and the surface area disappear, which is consistent with the analysis of XRD and N_2_ adsorption-desorption isotherms (Figure 2 and Figure 3b).

**Figure 6:**
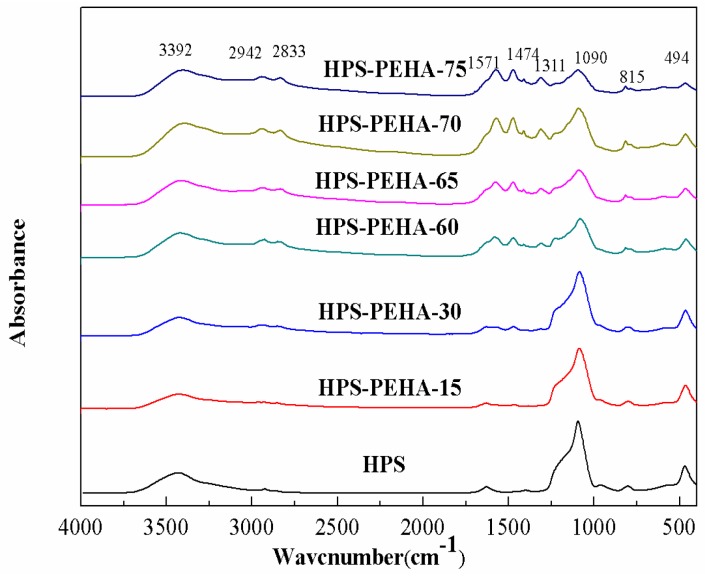
FT-IR spectra of the HPS and HPS-PEHA-x adsorbents.

Figure 6 shows the FT-IR analyses of the sorbents with different content of PEHA. Both the absorption bands at about 3392 cm^−1^ and 1695 cm^−1^ are attributed to hydrogen-bonded Si–OH and single Si–OH groups stretching vibrations, respectively [56,57]. With the PEHA loading increasing, the single Si–OH bond intensity gradually reduces and even disappears at the 30% PEHA loading, which implies that the PEHA could interact with the surface Si–OH groups. Such interaction could anchor the PEHA molecules on the silica surface, leading to increased sorbent thermal stability. The bands at 1571 cm^−1^ and 1474 cm^−1^ are attributed to the primary amines (–NH_2_) asymmetric and symmetric bending, respectively [58]. The bands at 1311 cm^−1^ are assigned to the C–N stretching modes of the PEHA chain. The bands at 2942 cm^−1^ and 2833 cm^−1^ are ascribed to the CH_2_ asymmetric and symmetric stretching modes [44,59]. The peaks intensity increase with the rise of the PEHA loading, which indicates that the mesopores and macropores of the silica are successfully incorporated in the PEHA.

**Scheme 2:**
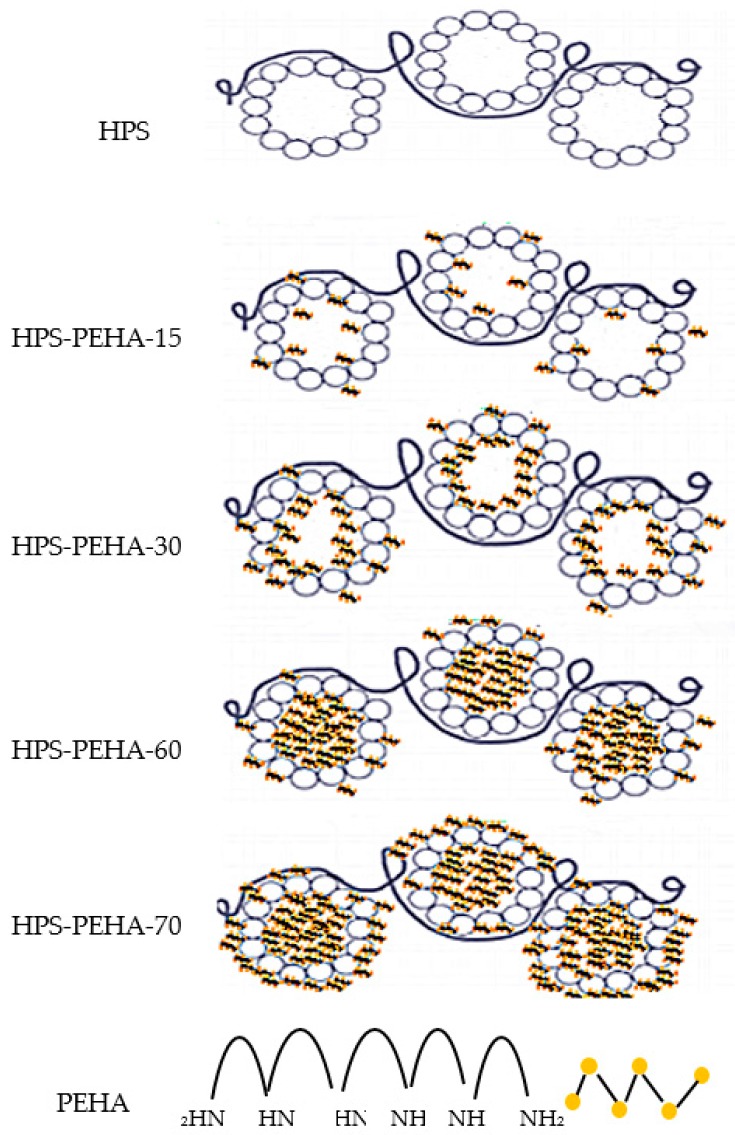
Schematic illustration of HPS and HPS-PEHA-x adsorbents.

A schematic diagram for PEHA distribution in HPS is depicted in Scheme 2. When the loading of PEHA is low, PEHA mainly scatters in the mesoporous (<30%). With decreasing mesoporous, PEHA mainly scatters in macropores until PEHA films fully cover the silica surface, which is consistent with the XRD, BET, SEM, and TEM results.

### 3.2. Effect of PEHA Loading on CO_2_ Adsorption

**Scheme 3:**
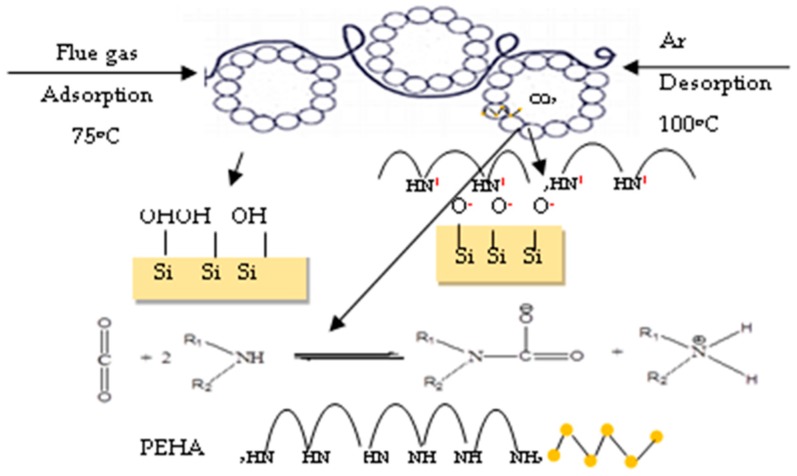
CO_2_ adsorption processes on PHS-PEHA-x adsorbents.

**Figure 7:**
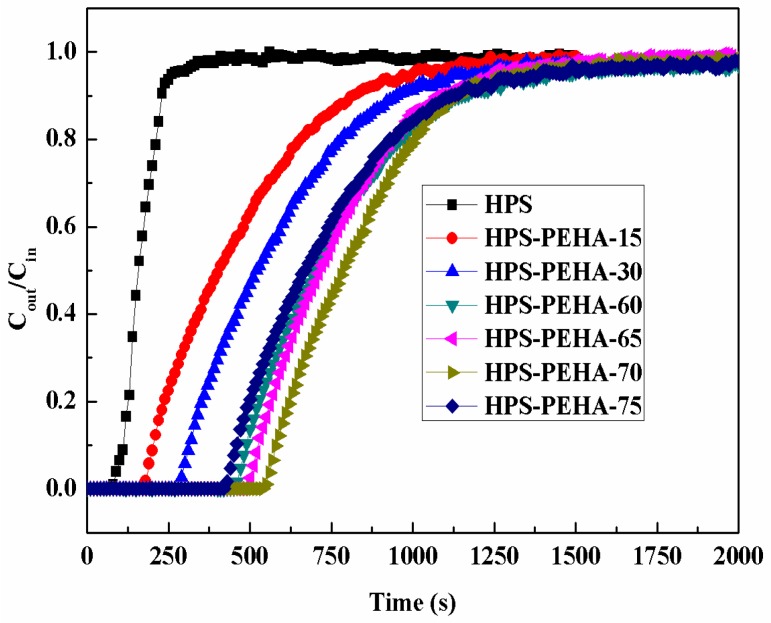
Breakthrough curves of CO_2_ against absorption time on HPS and HPS-PEHA-x adsorbents.

**Figure 8:**
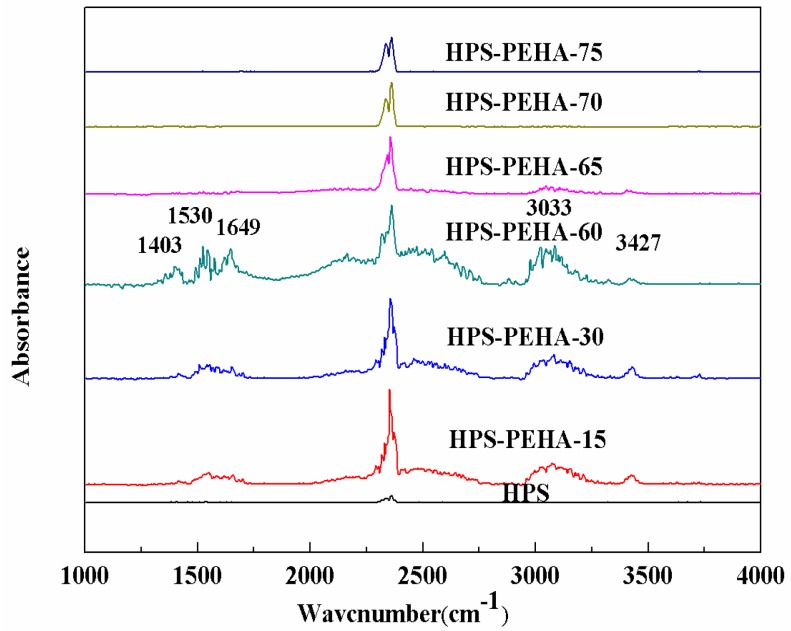
DRIFTS spectra of HPS and HPS-PEHA-x adsorbents.

Scheme 3 shows the CO_2_ adsorption performance for the adsorbents. CO_2_ adsorption capacities over HPS sorbents with various loadings of PEHA calculated from the breakthrough curves (Figure 7) are shown in Table 2. In-situ DRIFTS was also employed to study the adsorption mechanism over HPS-PEHA-x adsorbents (Figure 8). It can be found that upon introduction of CO_2_, absorption peaks at 3427, 3033, and 1649 cm^−1^ ascribed to N–H stretching vibration of RNHCOO^−^ and N–H expansion and bending vibration of RNH^3+^ appear, suggesting the formation of ammonium carbamate [60,61,62]. In addition, the peaks at 1530 cm^−1^ and 1403 cm^−1^ can be attributed to asymmetric and symmetric stretching vibration of O–C–O in the COO^−^ ion [63], which is similar to the result found in the literature [60,61,62]. With the HPS-support with HPS, the PEHA amine groups interact with the HPS surface silanol groups which cannot react with CO_2_. For HPS-PEHA-15, the majority of the amine groups are consumed with the action of the silanol group, which results in low CO_2_ adsorption capacity (1.4 mmol/g). The amine group amount which could interact with CO_2_ increased gradually, as the PEHA loading increased. When the amount of PEHA increased to 70%, the highest adsorption capacity of 4.5 mmol/g was obtained, higher than that of PEHA impregnated SBA-15 [44] or MCM-41 [64]. This may be due to the support material with large pore volume and hierarchical pore structures. Where the former is available with PEHA, loading increased, and the latter could be promoted to sorption sites and decrease the internal diffusion distance. However, the decrease of adsorption capacity for HPS-PEHA-75 might be due to the fact that the PEHA could fully fill the pore as was confirmed by N_2_ adsorption/desorption measurements. In order to analyze the influence of PEHA loading on the adsorption quantity, we can also introduce CO_2_-philic sites, which are determined through CO_2_/N mole ratios, to explain the influence in the process of CO_2_ adsorption-desorption [65,66]. As shown in Table 2, the percent of amine addition in HPS is increased from 15% to 75%. When the amount of PEHA increase to 70%, the highest adsorption capacity increases to 4.5 mmol/g. This may be due to the increase in the amount of CO_2_-philic adsorption sites. However, with the increase of PEHA loading to 75%, the adsorption capacity decreased to 3.7 mmol/g. It also can be explained by the amine efficiencies (CO_2_/N mole ratios). The CO_2_/N mole ratios decrease from 0.35 to 0.26. The reason is that excessive PEHA could fully fill the pore, confirmed by N_2_ adsorption/desorption measurements. This leads to the formation of a thick bulk PEHA layer and is accompanied by a reduction in the quantity of amine sites.

### 3.3. Effect of Adsorption Temperature on CO_2_ Adsorption

The CO_2_ adsorption capacities of HPS-PEHA-70 at temperatures ranging from 30–90 °C are displayed in Table 3. With the adsorption temperature increasing from 30 to 75 °C, the CO_2_ adsorption capacity increases from 3.2 to 4.5 mmol/g. Nevertheless, the CO_2_ adsorption capacity decreases significantly with further increased adsorption temperature. We can also introduce the two-amine-layer adsorption model (the inner bulky PEHA layer and the exposed PEHA layer) in the process of CO_2_ adsorption-desorption to analyze the impact [65,66]. At a relatively low temperature, the inner bulky PEHA layer plays the main role. The packed PEHA provides a low amine active site. However, as the temperature rises to 75 °C, the exposed PEHA layer plays a major role. High temperature causes organic amine stretching which leads to an increase in the number of exposed PEHA layers. This gives a good result in that amine sites and CO_2_ adsorption capacity increases. This can be explained well by the change CO_2_/N mole ratios. However, with further increase of the adsorption temperature, CO_2_ adsorption is mainly affected by the adsorption thermodynamics and CO_2_ desorption plays a dominant role. Consequently, the optimal temperature for CO_2_ adsorption thermodynamically and kinetically is 75 °C.

### 3.4. Regenerability of HPS-PEHA-70

**Figure 9:**
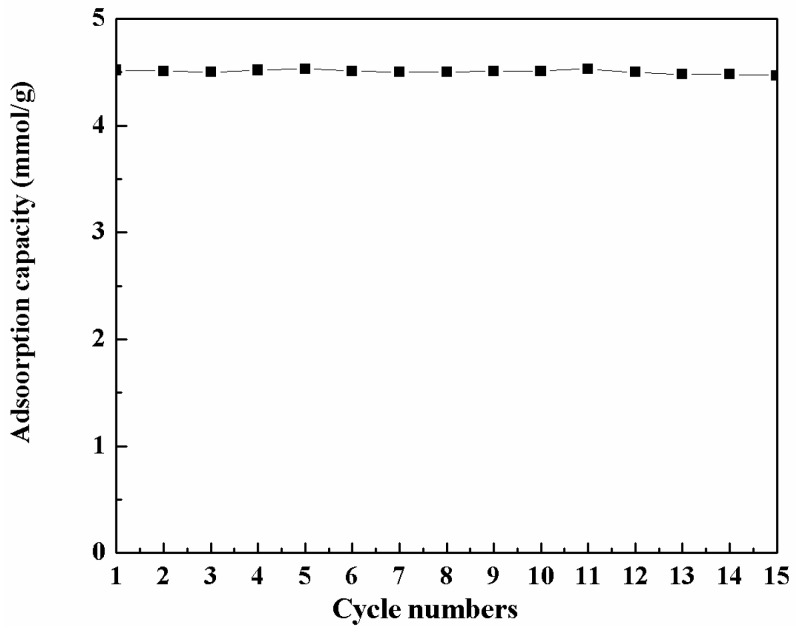
Recyclability of HPS-PEHA-70 sorbent.

The recyclability of the HPS-PEHA-70 sample is shown in Figure 9. The material is found to have stable adsorption capacity (with >96% desorption ability) even after 15 cycles, confirming the material to be ideal for CO_2_ adsorption.

### 3.5. Breakthrough Analysis Based on the Deactivation Model

Chemical adsorption of CO_2_ on amine-impregnated adsorbents can be considered as a non-catalytic reaction between fluid and solid. In the process of reaction, the surface of the solid phase reactant may form a dense layer of product which may lead to diffusion resistance and decline in the rate of gas-solid reaction. Moreover, the reaction may also cause changes in the active surface, pore structure, as well as the solid reactants activity. A deactivation model, considering the effect of these factors, can simulate the experimental data. Therefore, it requires a deactivation model containing two parameters to simulate the experimental data. These two parameters are the adsorption rate constant and deactivation rate constant, *k*_o_ and *k_d_*, respectively. The deactivation model is based on the following basic assumptions (1) the system is an isothermal bed; (2) the adsorption process is pseudo-steady-state; and (3) only the change of Z phase concentration (axial dispersion in the packed column and any mass transfer resistances are negligible) is considered.

The mass balance equation can be described as:

(1) $$-Q\frac{dC_{A}}{dW}-k_{0}C_{A}\alpha =0$$

Activity rate equation of solid phase adsorbents is as follows:

(2) $$-\frac{d\alpha }{dt}=k_{d}C_{A}^{n}\alpha^{m}$$

Adsorbent deactivation rate associated with adsorption gas reaction concentration, when *n* = 1, *m* = 1, can be described as:

(3) $$\frac{C_{A}}{C_{A0}}=\exp\left[\frac{1-\exp\left[\left(k_{0}W/Q\right)\left(1-\exp(-k_{d}t)\right)\right]}{1-\exp(-k_{d}t)}\exp(-k_{d}t)\right]$$

where, *C_A_* = concentration of reactant gas, mmol·cm^−3^; *k_d_* = deactivation rate constant, cm^3^·min^−1^·g^−1^; *k*_o_ = initial sorption rate constant, cm^3^·min^−1^·g^−1^; *a* = activity of the solid reactant; *Q* = volumetric flow rate, cm^3^·min^−1^; *t* = time, min; *W* = catalyst mass, g.

The parameters *k*_o_ and *k_d_* are obtained from regression analysis through nonlinear regression. The correlation coefficient R^2^ determines the correlation of the regression analysis.

The adsorption rate constant *k*_o_, deactivation rate constants *k_d_*, and correlation coefficient R^2^ obtained are shown in Table 2. For all sorbents, the deactivation equations fit the experimental breakthrough curves well and the correlation coefficients (R^2^) are all larger than 0.99, indicating that the deactivation model could correctly describe the adsorption process of the sorbents studied. It was found that with increasing PEHA loading, the kinetic rate constants *k*_o_ and *k_d_* increase dramatically, which could result from the CO_2_ short diffusion distance from the surface into the PEHA films bulk. It was found that *k*_o_ of HPS-PEHA-70 was significantly higher than conventional materials [67,68,69]. However, when the content of PEHA is higher than 75%, *k*_o_ decreases significantly and the value of *k_d_* increases. It might be because excessive organic amine may block most of the channel and lead to a large number of deactivated sites. The results fully correspond with their adsorption capacity and adsorption performance.

The experiments were also performed to study the temperature effect on the kinetics adsorption performance. It can be seen that the experimental data fits well with the deactivation model predictions in Table 3. It was found that *k*_o_ and *k_d_* increase as the temperature increased, suggesting that the CO_2_ diffusion and adsorption processes of HPS-PEHA-70 are enhanced. However, at 90 °C, *k_d_* increased dramatically, which may be more helpful in a stronger desorption process than an adsorption process.

## 4. Conclusions

In this work, porous silica material-supported PEHA with macropores and long-range ordering mesopores was prepared. The macropores could facilitate the sorption sites and decrease the internal diffusion distance, and the mesopores were available for increasing the PEHA loading and dispersion. The synergetic effects of the two kinds of pores greatly improved the CO_2_ adsorption. It was found that the temperature and the loading of PEHA amount on the porous silica play an important role in the sorbents sorption behavior. CO_2_ adsorption capacity of 4.5 mmol of CO_2_/(g sorbent) could be obtained at a temperature of 75 °C for HPS-PEHA-70. Moreover, the sorption has good stability. After a total of 15 adsorption-desorption cycles, the adsorption capacity for HPS-PEHA-70 remains steady.

## Figures and Tables

**Table 1:** Pore structure parameters of the HPS and HPS-PEHA-x adsorbents.

Samples	S_BET_ (m^2^/g)	V_BJH_ (cm^3^/g)	V_mic_ (cm^3^/g)	D_average_ (nm)
HPS	544	1.00	0.03	6.96
HPS-PEHA-15	302	0.60	0	7.59
HPS-PEHA-30	89	0.34	0	15.04
HPS-PEHA-60	7	0.02	0	23.90
HPS-PEHA-65	2	0	0	7.60
HPS-PEHA-70	0	0	0	0
HPS-PEHA-75	0	0	0	0

**Table 2:** Adsorption capacity of CO_2_ over HPS and HPS-PEHA-x adsorbents.

Sample	Adsorption Temperature (°C)	N Content (mmol/g)	Adsorption Capacity (mmol/g)	CO_2_/N	*k*_o_ (mL/min·g)	*k_d_* (min^−1^)	R^2^
HPS	75	0	0.3	–	150.08	0.37	0.9989
HPS-PEHA-15	75	3.68	1.4	0.38	752.21	0.40	0.9987
HPS-PEHA-30	75	6.67	2.4	0.36	1042.95	0.45	0.9977
HPS-PEHA-60	75	10.80	3.8	0.35	1107.87	0.47	0.9989
HPS-PEHA-65	75	12.06	4.2	0.35	1175.99	0.47	0.9988
HPS-PEHA-70	75	13.03	4.5	0.35	1243.28	0.49	0.9986
HPS-PEHA-75	75	14.38	3.7	0.26	910.68	0.70	0.9977

**Table 3:** CO_2_ Adsorption capacities of HPS-PEHA-70 at various adsorption temperatures.

Sample	Adsorption Temperature (°C)	N Content (mmol/g)	Adsorption Capacity (mmol/g)	CO_2_/N	*k*_o_ (mL/min·g)	*k_d_* (min^−1^)	R^2^
HPS-PEHA-70	30	13.03	3.2	0.26	806.35.	0.19	0.9997
	50	13.03	3.7	0.28	951.43	0.31	0.9995
	70	13.03	4.3	0.33	1292.67	0.45	0.9995
	75	13.03	4.5	0.35	1343.28	0.49	0.9994
	90	13.03	3.5	0.27	1798.18	0.99	0.9991
